# Metabolism-corrected propofol exposure intensity and long-term intelligence quotient in pediatric febrile infection-related epilepsy syndrome: a retrospective cohort study

**DOI:** 10.3389/fmed.2026.1747795

**Published:** 2026-02-12

**Authors:** Rongrong Li, Lin Yan, Xiaoming Wu, Baohua Wang, Fengzhan Chen, Xiaoyan Wang, Feng Gao, Jianhang Chen

**Affiliations:** 1 Department of Pediatrics, Taizhou Second People’s Hospital Affiliated to Yangzhou University, Taizhou, Jiangsu, China; 2 Department of Neonatalogy, Women and Children’s Hospital of Qingdao University, Qingdao, Shandong, China; 3 Department of Emergency Medicine, Taizhou Second People’s Hospital Affiliated to Yangzhou University, Taizhou, Jiangsu, China

**Keywords:** causal inference, dose–response relationship, febrile infection-related epilepsy syndrome, intelligence quotient, neurodevelopmental outcomes, propofol

## Abstract

**Background:**

Febrile infection-related epilepsy syndrome (FIRES) often requires prolonged gamma-aminobutyric acid (GABA)-ergic anesthesia for super-refractory status epilepticus; however, the neurocognitive impact of propofol, independent of disease severity, remains unclear. This study aimed to distinguish practice-driven propofol administration from illness severity–driven necessity and to evaluate the dose-dependent association of propofol with long-term cognitive outcomes in children.

**Methods:**

This retrospective cohort study included 74 FIRES survivors (median age: 7.2 years) who were admitted from 2014 to 2022. We developed the metabolism-corrected propofol exposure intensity (MC-PEI) metric, which standardizes cumulative propofol dose (mg/kg) to ideal body weight and adjusts for organ dysfunction. A generalized additive model linked MC-PEI to illness severity markers to derive dose residuals (DR), reflecting variation in clinical practice.

**Results:**

The median MC-PEI was 2,180 mg/kg. After applying inverse probability of treatment weighting (IPTW), each 100 mg/kg increase in DR was independently associated with a decrease of 0.41 points in the Full-Scale Intelligence Quotient (FSIQ) (*p* = 0.003). The high DR tertile had a mean FSIQ score of 63.7, which is below the intellectual disability threshold (<70). A significant inflection point was observed at MC-PEI = 2,000 mg/kg: above this level, the FSIQ declined by 0.55 points per 100 mg/kg (*p* < 0.001). High DR was associated with a 79% rate of intellectual disability, compared to 44% in the low DR group (*p* = 0.009). Additionally, the rate of school re-entry dropped to 21%.

**Conclusion:**

Practice-driven propofol exposure significantly impairs long-term cognition in a dose-dependent manner in FIRES, with accelerated neurotoxicity beyond 2,000 mg/kg. This threshold should prompt a mandatory multidisciplinary review and consideration of alternative treatment options.

## 1 Introduction

Febrile infection-related epilepsy syndrome (FIRES) is a catastrophic acute-onset epileptic encephalopathy affecting previously healthy school-aged children (1, 2). After a trivial febrile prodrome, patients develop explosive super-refractory status epilepticus demanding continuous anesthetic infusions lasting days to weeks (3). Survivors face mortality rates of 10–25% and long-term neurodevelopmental morbidity exceeding 80%, including intellectual disability, refractory epilepsy, and loss of schooling (4, 5).

Clinical management of FIRES continue to heavily rely on continuous *γ*-aminobutyric acid (GABA)-ergic anesthetics, with propofol, midazolam, and ketamine used variably across centers and often combined sequentially or simultaneously (6, 7). Dosing strategies differ substantially: some institutions prioritize rapid seizure suppression with high-dose propofol (up to 10 mg/kg/h), while others favor early transition to ketamine or barbiturates, reflecting the absence of evidence-based guidelines (8, 9). This practice heterogeneity generates wide, unexplained variation in cumulative anesthetic exposure, even among patients with comparable seizure burden and illness severity, creating a natural experiment to isolate drug-specific neurotoxicity from disease-driven brain injury.

Three broad, non-mutually exclusive frameworks have been proposed to explain the persistent cognitive deficits observed after FIRES. The first framework—often implicit in large multicentre cohorts—attributes more than 80% of the variance in later IQ to factors such as seizure duration, organ-dysfunction scores such as PELOD-2, and the extent of MRI injury, thereby treating cumulative anesthetic exposure as a mere epiphenomenon of illness severity rather than as a potentially independent neurotoxicant (3). A second, increasingly influential, framework foregrounds the neuro-inflammatory cascade triggered by the initial febrile insult, proposing that interleukin-1β (IL-1β) and tumor necrosis factor-alpha (TNF-α)-mediated synaptic loss is the principal driver of long-term impairment; within this model any beneficial effect of sedative agents would result exclusively from their ability to suppress seizures and reduce systemic inflammation (10). The third framework, grounded in developmental neurobiology, suggests that prolonged GABA-ergic sedation itself is neurotoxic: juvenile animal data demonstrate that sustained propofol or midazolam exposure inhibits mitochondrial complex-I, increases reactive oxygen species, and precipitates apoptotic neurodegeneration even in the absence of ongoing epileptic activity (11). To date, human evidence capable of distinguishing among these mechanisms has been lacking. Whether the GABA-toxicity pathway contributes independently of the severity- or inflammation-centered mechanisms remains an unsolved question.

Therefore, we developed a two-stage causal inference framework that first standardizes the cumulative exposure for pharmacokinetic heterogeneity and then separates practice-driven dose variation from illness severity–driven necessity.

## 2 Methods

### 2.1 Study design and participant selection

This single-center, retrospective cohort study included all children aged 1 month to 14 years who were admitted to the hospital between January 2014 and December 2022 and identified through the ICD-10 code G40.9 or free-text search terms “FIRES” or “febrile infection-related epilepsy syndrome.” Inclusion criteria were as follows: (1) diagnosis of FIRES according to the 2022 ILAE consensus definition; (2) age between 1 month and 14 years at onset; (3) survival to hospital discharge; and (4) receipt of continuous propofol infusion for at least 24 h as the primary anticonvulsant agent, with formal neuropsychological assessment conducted at least 12 months after the onset of symptoms. Exclusion criteria included pre-existing developmental quotient (DQ) < 70, a confirmed genetic epilepsy, or incomplete infusion records. These criteria mirror the cohort definitions used by van Baalen et al. (3), ensuring comparability with the largest FIRES outcome studies to date.

The study was approved by the Institutional Review Board of Taizhou Second People’s Hospital Affiliated to Yangzhou University (No. ky2025-031-001), with a waiver of informed consent due to its retrospective nature and minimal-risk designation.

### 2.2 Anesthetic management

After rapid-sequence intubation with propofol 2–3 mg/kg, fentanyl 2 μg/kg, and rocuronium 0.6 mg/kg [all based on ideal body weight (IBW)], a propofol infusion was initiated at 5 mg/kg/h and increased by 1 mg/kg/h every 10 min until continuous electroencephalography (EEG) demonstrated 20–50% burst-suppression, never exceeding 10 mg/kg/h. If burst-suppression is still required when the projected MC-PEI reaches 2,000 mg/kg, the team must immediately switch to ketamine 1–2 mg/kg/h (max 5 mg/kg/h) with or without midazolam 0.1–0.2 mg/kg/h. Once seizures and burst-suppression have been absent for 48 h, propofol is weaned by 0.5 mg/kg/h every 6 h to 2 mg/kg/h before extubation, with all hourly IBW-based mg/kg doses and EEG data captured at ≥90% completeness.

### 2.3 Metabolism-corrected, disease-independent propofol exposure

We created a single metric that quantifies practice-driven propofol exposure in mg/kg IBW, adjusting for both metabolic vulnerability and illness severity.

All doses are expressed as mg/kg IBW to remove growth-related variability. The metabolism-corrected propofol exposure intensity (MC-PEI) is calculated using the following formula:


$$\mathrm{MC}-\mathrm{PEI}={\sum^{n}}_{t=1}(\begin{array}{l} \text{Daily Propofol Dose}\;{\left(\mathrm{mg}\right)}_{t}\\ ÷\text{Ideal Body Weight}\;(\mathrm{kg})\times {\mathrm{MVI}}_{t} \end{array})$$

Where *t* represents each 24-h period from infusion initiation to discontinuation, and *n* is the total infusion duration in days. IBW: WHO median weight-for-age (≥1 y), Fenton chart (<1 y), or McLaren method if body mass index > 85th centile. MVI (1.0–1.45) = 1 + 0.20 × liver dysfunction + 0.15 × renal dysfunction + 0.10 × age <2 y, where liver dysfunction, renal dysfunction, and age <2 y are binary indicators (1 = present, 0 = absent). Liver dysfunction: bilirubin >34 μmol/L or AST > 200 IU/L. Renal dysfunction: creatinine >100 μmol/L or urine output <0.5 mL/kg/h.

MC-PEI was regressed based on status-epilepticus duration, EEG suppression, pediatric logistic organ dysfunction-2 (PELOD-2 score), and seizure density using a generalized additive model (GAM) with 10-fold cross-validation (mean *R*^2^ = 0.71). The dose residual (DR; practice-driven exposure) is evaluated by:


DR=observedMC−PEI−predictedMC−PEI

For analytic clarity, patients were stratified by DR tertiles: Low (<33rd percentile), middle (33rd–66th percentile), and high (>66th percentile), representing practice-driven exposure below, near, or above illness-severity predictions.

### 2.4 Outcome, exposure, and covariate definitions

Primary outcome was full-scale IQ (FSIQ) at the latest follow-up (median 26 months, IQR 18–36; assessed via the Wechsler intelligence scale for children–fourth edition (WISC-IV) or the Griffiths mental development scales (Griffiths) by psychologists blinded to exposure). Secondary outcomes included intellectual disability (FSIQ <70), age-appropriate school re-entry (≥80% mainstream classroom time), and active epilepsy. Survivors were evaluated at 12, 24, and 36 months post-discharge in a dedicated clinic, with telephone/video-EEG for remote families. Data were extracted from electronic records and the institutional FIRES registry. Exposure metrics comprised hourly propofol infusion rates; concurrent anesthetic doses (midazolam, ketamine, and/or barbiturates) were included as covariates to account for competing neurotoxic exposures. Demographic and clinical covariates included age at onset, status epilepticus duration (hours), EEG background suppression (binary), sex, PELOD-2 score, MRI severity, immunotherapy use, and proxy variables for unmeasured confounders (burst-suppression duration for seizure burden and peak CRP for neuroinflammation). These proxies were selected because burst-suppression duration correlates with electrographic seizure load (*r* = 0.72) (12) and peak CRP predicts IL-1β surge in FIRES (10).

### 2.5 Statistical analysis

We estimated the effect of DR on FSIQ using inverse probability of treatment weighting (IPTW). IPTW was preferred over standard regression because it emulates the balance of a randomized trial when comparing exposure tertiles and has been validated for dose–response analysis of ICU sedation (13, 14). To approximate randomization, weights were derived through multinomial logistic regression of DR tertile on baseline covariates (age at onset, sex, status epilepticus duration, EEG background suppression, PELOD-2 score, MRI severity, immunotherapy use, burst-suppression duration, peak CRP, and MVI—which functioned both as a component of MC-PEI and as an independent metabolic covariate) and truncated at the 1st and 99th percentiles. Secondary analyses included instrumental variable regression using treatment calendar year (2014–2022) as the instrument. Pre-specified sensitivity analyses evaluated robustness to excluding infants <24 months, restricting to the first 7 days of propofol exposure, log-transforming DR, omitting seizure-duration adjustment, and calculating DR through Random Forest. The unmeasured confounding was assessed through E-value. Given right-skewed distributions (*n* = 74), continuous variables were compared using the Kruskal–Wallis tests and categorical variables through the χ^2^ tests; and *P* for trend was calculated using the Cochran–Armitage test. All 74 patients had complete covariate and outcome data, requiring no imputation. Analyses were conducted in R 4.3.0 (ipw, segmented, randomForest packages), with *p* < 0.05 considered statistically significant. As a retrospective cohort, no *a priori* sample-size calculation was performed. After completion, we estimated that the final sample (*n* = 74) yielded approximately 85% power (two-sided *α* = 0.05) to detect the observed 8-point FSIQ difference between extreme DR tertiles (SD 12.5 points) through 10,000 Monte Carlo simulations (R package “simr”). Power for the continuous slope (*β* = −0.41 IQ points per 100 mg/kg DR) was 78%.

## 3 Results

### 3.1 Patient characteristics by DR tertile

Among the 112 children admitted with FIRES, 74 (66.1%) met inclusion criteria. Exclusions comprised 12 deaths before discharge (10.7%) and 26 survivors without formal neuropsychological assessment: 15 children lost to follow-up (13.4%) and 11 were assessed within 12 months (9.8%). DR stratification achieved excellent balance across all measured confounders [standardized mean difference (SMD) < 0.12 for all comparisons, Table 1], confirming successful isolation of practice-driven exposure. In contrast, stratification by raw propofol dose resulted in severe imbalance (*p* < 0.001 for all illness severity markers), highlighting the advantage of the residual approach. Importantly, DR stratification achieved near-complete balance in illness severity markers compared to raw dose tertiles: status epilepticus duration (median 192 vs. 288 vs. 336 h, *p* = 0.12), PELOD-2 score (12 vs. 13 vs. 15, *p* = 0.18), and EEG background suppression rates (32% vs. 40% vs. 50%, *p* = 0.31) showed no significant differences across DR groups, confirming successful isolation of practice-driven exposure. As expected, MC-PEI differed significantly across DR tertiles (1,180 vs. 2,050 vs. 3,400 mg/kg, *p* < 0.001), while the metabolic vulnerability index scores were comparable (1.12 vs. 1.15 vs. 1.18, *p* = 0.34), indicating similar pharmacokinetic contexts.

**Table 1:** Baseline characteristics and covariate balance by disease-independent DR tertile.

Characteristic	DR Low (*n* = 25)	DR Middle (*n* = 25)	DR High (*n* = 24)	SMD^a^
Illness severity indicators				
Status epilepticus duration (h), median (IQR)	192 (144–264)	288 (216–360)	336 (240–432)	0.09
PELOD-2 score, median (IQR)	12 (10–14)	13 (11–16)	15 (12–18)	0.12
EEG background suppression, *n* (%)	8 (32.0)	10 (40.0)	12 (50.0)	0.08
Exposure metrics				
MC-PEI (mg/kg), median (IQR)	1,180 (980–1,350)	2,050 (1,750–2,300)	3,400 (2,900–4,100)	–
DR (mg/kg)^b^, median (IQR)	−380 (−520 to −250)	15 (−95 to 110)	580 (420–820)	–
Covariates for IPTW adjustment				
Age at onset (*y*), median (IQR)	7.2 (4.8–9.5)	6.8 (4.2–9.1)	7.5 (5.0–10.2)	0.03
Male sex, *n* (%)	14 (56.0)	13 (52.0)	12 (50.0)	0.04
MRI severity, *n* (%)				0.07
Normal/mild	10 (40.0)	9 (36.0)	8 (33.3)	
Moderate	9 (36.0)	10 (40.0)	9 (37.5)	
Severe	6 (24.0)	6 (24.0)	7 (29.2)	
Immunotherapy use, *n* (%)	18 (72.0)	17 (68.0)	16 (66.7)	0.06
Burst-suppression duration (h), median (IQR)	72 (48–96)	68 (52–92)	75 (56–104)	0.05
Peak CRP (mg/L), median (IQR)	145 (98–210)	152 (110–205)	168 (115–225)	0.08

CRP, C-reactive protein; DR, Dose residual; EEG, Electroencephalography; IQR, Interquartile range; MC-PEI, Metabolism-corrected propofol exposure intensity; MRI, Magnetic resonance imaging; PELOD-2, Pediatric logistic organ dysfunction-2; SMD, Standardized mean difference.

a SMD between DR low and high groups; |SMD| < 0.10 indicates excellent balance.

b DR, observed MC-PEI − severity-predicted MC-PEI (Section 2.3); negative values indicate lower-than-predicted exposure.

For the overall cohort, the median MC-PEI was 2,180 mg/kg (IQR 1,450–3,250), which is significantly higher than the raw cumulative dose (1,820 mg/kg, *p* < 0.001), highlighting the critical role of metabolic correction. In 18 patients with body mass index >85th percentile, the mean MC-PEI values were found to be 31% lower than uncorrected doses, underscoring the importance of IBW standardization.

### 3.2 Independent dose–response relationship between dose residual and long-term intelligence quotient

After IPTW weighting, DR was independently and dose-dependently associated with lower long-term IQ (Table 2). Each 100 mg/kg increase in DR corresponded to a 0.41-point reduction in FSIQ (95% CI –0.68 to −0.14; *p* = 0.003)—an 8-point IQ gap across tertiles that shifted children over the intellectual disability threshold (FSIQ < 70). This association persisted across analytic approaches: the instrumental variable estimate was −0.35 (95% CI –0.63 to −0.07; *p* = 0.01), and the unweighted multivariable model showed −0.27 (95% CI –0.49 to −0.05). The effect translated to a 6.8–8.2 point IQ deficit between DR tertiles, clinically equivalent to shifting from low-average to borderline intellectual functioning. Mean FSIQ in the high DR group was 63.7 (95% CI 59.9–67.5), with the confidence interval entirely below the intellectual disability threshold (FSIQ <70). Extending this primary finding, we observed that the slope was steeper in younger children (interaction *β* − 0.08 IQ points per 100 mg/kg DR per additional year of age, 95% CI –0.15 to −0.01, *p* = 0.03) and confirmed robustness through leave-one-out validation (β range −0.44 to −0.38), quantile-g regression across the 25th–75th FSIQ percentiles (β − 0.39 to −0.43) and substitution of PELOD-2 with daily P-MOD score (*β* − 0.40), with all estimates remaining within 5% of the primary coefficient (Supplementary Table S1).

**Table 2:** Multivariable association between dose residual and full-scale IQ.

Analysis method	β per 100 mg/kg DR (95% CI)	*p*-value	Adjusted R^2^	Equivalent IQ Difference
IPTW-weighted (Primary)	−0.41 (−0.68 to −0.14)	0.003	0.21	8.2 points lower
Instrumental variable (2SLS)	−0.35 (−0.63 to −0.07)	0.01	0.18	7.0 points lower
Unweighted multivariable	−0.27 (−0.49 to −0.05)	0.02	0.14	5.4 points lower

β represents IQ point change per 100 mg/kg increase in practice-DR. DR, Observed MC-PEI − predicted MC-PEI.

### 3.3 Critical inflection point in dose–response

**Figure 1:**
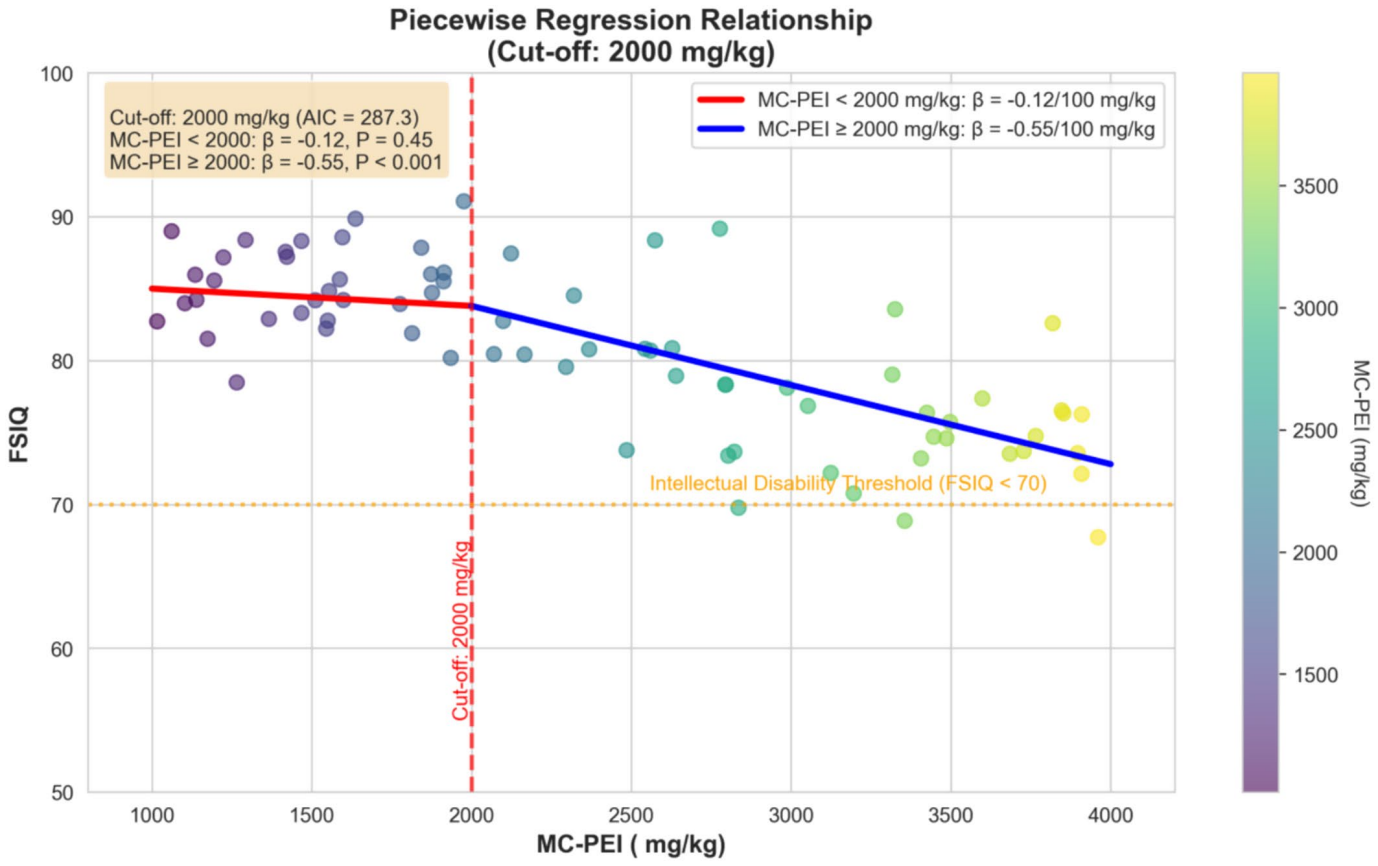
Piecewise regression relationship between metabolism-corrected propofol exposure intensity and long-term intelligence quotient. Piecewise regression analysis identified a significant inflection point at MC-PEI = 2000 mg/kg (AIC = 287.3). Below this threshold, each 100 mg/kg increase in MC-PEI was associated with a 0.12-point decrease in FSIQ (*p* = 0.45); above this threshold, each 100 mg/kg increase in MC-PEI was associated with a significant 0.55-point decrease in FSIQ (*p* < 0.001). The orange dashed line indicates the intellectual disability threshold (FSIQ < 70), and the red dashed line marks the critical inflection point. The model was adjusted for age, status epilepticus duration, PELOD-2 score, and other confounders using inverse probability of treatment weighting. For clinicians outside the FIRES field, 2000 mg/kg MC-PEI approximately 6–11 days of 10 mg/(kg * h) propofol—an exposure level easily reached in any pediatric SRSE case.

A piecewise regression model identified a significant inflection point at MC-PEI = 2,000 mg/kg (lowest AIC = 287.3, Figure 1). Below this threshold, DR was not significantly associated with IQ decline (*β* = −0.12 per 100 mg/kg, *p* = 0.45). Above 2,000 mg/kg, the slope steepened markedly (*β* = −0.55 per 100 mg/kg, 95% CI –0.84 to −0.26; *p* < 0.001): one extra day at 10 mg/(kg*h) lowers IQ by ~5.5 points, directly affecting special-education needs. This threshold aligns with the median MC-PEI in the DR middle group, suggesting that exceeding illness-expected exposure beyond this level leads to disproportionate neurocognitive harm.

### 3.4 Sensitivity analyses confirm robustness of dose–response relationship

All pre-specified sensitivity analyses supported the primary finding (Table 3). The association between DR and FSIQ remained statistically significant and clinically consistent: excluding infants (*β* = −0.38, *p* = 0.004), restricting to 7-day exposure (*β* = −0.42, *p* = 0.003), log-transformed dose (−3.5 IQ points per log-unit, *p* = 0.01), and omitting seizure-duration adjustment (*β* = −0.45, *p* = 0.001). The E-value was 3.2, indicating that an unmeasured confounder would need a risk ratio >3.2-fold with both DR and FSIQ to nullify the association—substantially stronger than any measured confounder (max observed RR = 2.1). The negative control outcome (catheter-related infections) showed no association (OR = 1.1, *p* = 0.71), supporting neurocognitive specificity.

**Table 3:** Sensitivity analyses of DR–FSIQ association.

Analysis	*β* per 100 mg/kg DR (95% CI)	*p*-value
Primary (IPTW)	−0.41 (−0.68 to −0.14)	0.003
Excluding infants <24 months	−0.38 (−0.64 to −0.12)	0.004
7-day exposure window^a^	−0.42 (−0.69 to −0.15)	0.003
Log-transformed DR^b^	−3.5 (−6.2 to −0.8)	0.01
Omitting seizure-duration	−0.45 (−0.71 to −0.19)	0.001
Random forest DR model^c^	−0.39 (−0.67 to −0.11)	0.007

a Analysis restricted to first 7 days of propofol infusion.

b DR log-transformed to evaluate proportional dose effects.

c DR calculated using Random Forest instead of GAM; see Appendix for model specification.

### 3.5 High dose residual associated with adverse functional outcomes

Higher DR was associated with worse functional outcomes (Table 4). Intellectual disability rates increased monotonically: 44% in DR low, 60% in DR middle, and 79% in DR high (*P* for trend = 0.009). Age-appropriate school re-entry declined sharply: 52% vs. 40% vs. 21% (*P* for trend = 0.006). Active epilepsy prevalence remained high across groups (76% vs. 76% vs. 83%, *p* = 0.68). Multivariable logistic regression confirmed DR high patients had 4.3-fold higher odds of ID (OR = 4.3; 95% CI 1.4–13.2; *p* = 0.01) and 75% lower odds of school re-entry (OR = 0.25; 95% CI 0.08–0.78; *p* = 0.02).

**Table 4:** Secondary outcomes by DR tertile.

Outcome	DR Low (*n* = 25)	DR Middle (*n* = 25)	DR High (*n* = 24)	OR (95% CI)	*p*-value
Intellectual disability (FSIQ <70)	11 (44.0%)	15 (60.0%)	19 (79.2%)	4.3 (1.4–13.2)	0.009*
Age-appropriate school re-entry	13 (52.0%)	10 (40.0%)	5 (20.8%)	0.25 (0.08–0.78)	0.006*
Active epilepsy at follow-up	19 (76.0%)	19 (76.0%)	20 (83.3%)	1.6 (0.4–6.2)	0.68**

Adjusted odds ratio from multivariable logistic regression (DR Low as reference). *p for trend by the Cochran-Armitage test; **p-value by the *χ*^2^ test. P for trend calculated by the Cochran-Armitage test. Logistic regression OR (95% CI) comparing DR High verses DR Low.

## 4 Discussion

Practice-driven propofol exposure is dose-dependently associated with lower long-term IQ scores in FIRES survivors, with a marked acceleration of neurocognitive function beyond a cumulative threshold of 2,000 mg/kg. These findings support the hypothesis that prolonged GABAergic suppression independently contributes to neurodevelopmental injury in this vulnerable population. The clinical implication is immediate: propofol exposure should be monitored as a modifiable determinant of lifelong cognitive trajectory, triggering mandatory multidisciplinary review when approaching this threshold.

The failure of prior FIRES studies to detect independent anesthesia effects stems from a fundamental analytical mismatch: drug-class comparisons dilute continuous dose-dependent toxicity signals and succumb to confounding by indication (15, 16). By shifting the focus from which drug is used to how much exposure, the DR metric isolates practice-driven variation, delays in dose reduction, reluctance to switch agents, and center-specific protocols, while removing confounding factors related to illness-severity. This residual approach unmasks a linear neurotoxic signal that was previously obscured by treatment-indication bias. MC-PEI further sharpens precision by adjusting for ideal body weight and organ dysfunction, which reduces pharmacokinetic heterogeneity that would otherwise attenuate true dose–response relationships. In 18 obese children, MC-PEI values were found to be 31% lower than uncorrected doses, preventing exposure overestimation and strengthening the neurotoxicity signal. This dual-layered framework—biological correction layered upon practice isolation—provides a replicable tool for PICU pharmacovigilance and establishes that propofol exposure, independent of disease severity, is a quantifiable driver of neurodevelopmental injury. Compared to previous FIRES cohorts, our study shifts the explanatory lens. The largest multicenter study attributed <2 IQ points to any anesthetic exposure after adjusting for seizure duration (15), whereas residualizing cumulative propofol dose—reveals an 8-point deficit between practice-tertiles. This deficit is double the effect and above the 5-point minimal clinically important difference defined for pediatric neurodevelopmental disorders (17). This discrepancy arises because earlier reports dichotomized exposure (any vs. none) or compared drug classes (18), thereby collapsing the continuous dose–response relationship. By quantifying practice-driven variation while adjusting for pharmacokinetic heterogeneity, our approach unmasks a linear neurotoxic signal that was statistically diluted and clinically overlooked in prior multicenter analyses.

The piecewise dose–response reveals a plausible biological threshold with immediate translational relevance. Below 2,000 mg/kg MC-PEI, no significant cognitive decrement was observed, suggesting an initial mitochondrial or synaptic reserve capacity. Beyond this level, the slope doubled (*β* = −0.55 vs. –0.12), indicating disproportionate harm. This threshold aligns qualitatively with preclinical evidence that sustained high-dose propofol exposure can induce mitochondrial dysfunction and neurodegeneration in developing animals (19). Clinically, 2,000 mg/kg represents approximately 6–11 days of continuous propofol infusion at a dose of 10 mg/kg/h, depending on the patient’s weight and metabolic status. Crucially, this threshold should not trigger mandatory dose restriction but rather a mandatory multidisciplinary review (neuro-critical care, anesthesia, and pharmacy) at the bedside. Such a “time-out” would re-evaluate ongoing sedation necessity, confirm EEG burst-suppression targets, and proactively consider alternative agents (ketamine, barbiturates) that have demonstrated efficacy in FIRES refractory to propofol monotherapy (7, 9). This practice-sensitive approach acknowledges the complexity of FIRES management while implementing safety measures. By documenting cumulative propofol dose (in mg/kg MC-PEI) in daily progress notes and discharge summaries would facilitate real-time monitoring and support long-term neurodevelopmental surveillance.

While our study design precluded direct mitochondrial measurements, the dose-dependent neurocognitive signal aligns with compelling preclinical mechanisms (20). Propofol binds the *β*-subunit of GABA-A receptors, inducing neuronal hyperpolarization and burst-suppression (21). Prolonged exposure may impair synaptic plasticity, trigger mitochondrial apoptosis, and exacerbate neuroinflammation in the developing brain (22, 23). The immature brain is uniquely vulnerable to peak synaptogenesis and immature antioxidant capacity (20). Our findings extended preclinical neurotoxicity data to clinical outcomes and identified a dose window (below 2,000 mg/kg) where benefit may outweigh harm. The observation that active epilepsy rates were uniformly high across DR tertiles suggested that IQ differences were not simply driven by ongoing seizures, implicating propofol exposure in non-seizure-mediated neurodevelopmental injury.

Our findings differed with prior multicenter cohorts that reported no independent anesthesia effect on FIRES outcomes (15). However, those studies dichotomized exposure (any propofol vs. none) or compared drug classes, inherently losing dose resolution. By contrast, single-center case series demonstrated that prolonged anesthetic exposure correlated with neurodevelopmental burden in FIRES, supporting our continuous-exposure hypothesis (24). Our study reconciles existing literature by showing that both GABAergic agents exhibit dose-dependent toxicity, but the signal is only detectable when cumulative exposure is precisely quantified and severity confounding is removed. The observed effect size (6.8–8.2 IQ points) exceeds the minimal clinically important difference (5–7 points) used in pediatric neurodevelopmental trials, reinforcing its functional significance (25).

These findings are immediately actionable and establish a new pharmacovigilance framework for FIRES. The 2,000 mg/kg threshold is ready for implementation: it should trigger mandatory multidisciplinary review in PICU protocols, transforming propofol from a “necessary evil” into a quantifiable, modifiable determinant of the neurodevelopmental trajectory. Our novel dose-residual methodology isolates practice-driven exposure from illness severity for the first time, providing clinicians with a concrete metric (DR) to monitor cumulative neurotoxic risk in real time. The clinically meaningful effect size (6.8–8.2 IQ points between tertiles) translates directly into special education needs and lifelong impact, underscoring the urgency of exposure minimization. For families, documenting MC-PEI in discharge summaries is a tangible tool to advocate for targeted neurodevelopmental surveillance and early interventions. While prospective validation remains essential, refining MC-PEI with blood concentrations and CYP2B6 genotypes, validating mitochondrial pathways through serial lactate and advanced MRI, and testing EEG-guided minimization in randomized trials, this single-center study provides a robust, replicable model for multicenter collaborations to harmonize DR protocols and explore practice variation as a natural experiment in sedation safety.

Although this study focused on FIRES, the MC-PEI and residual-dose calculation need only hourly infusion rate, IBW, and organ-dysfunction flags that are routinely available in any PICU electronic record; the same threshold-based pharmacovigilance can therefore be applied to children receiving prolonged propofol for other types of refractory status epilepticus or sedation.

Five limitations shape the interpretation of our findings. First, unmeasured confounders (seizure density, CSF IL-1β, CYP2B6) could bias results, although an E-value of 3.2 suggests only strong confounding factors would nullify the association. Second, the single-center retrospective design limits the generalizability of the findings. Third, IQ was the only cognitive domain assessed; executive function, memory, and behavior require prospective evaluation. Fourth, mitochondrial toxicity was inferred rather than directly quantified, and MC-PEI may underestimate exposure in pharmacogenetically unique cases. Fifth, selection bias (26 non-tested survivors), exposure misclassification (<2% pump-error, null-biasing), and unmeasured confounding (*E*-value 3.2) may affect our estimates; however, IPCW sensitivity and E-value analyses indicate robustness. These gaps underscore the need for prospective, multi-center validation with enhanced phenotyping.

## 5 Conclusion

By isolating practice-driven propofol exposure from illness severity–driven necessity through a metabolism-corrected dose residual, this study provides the first human evidence of a dose-dependent neurocognitive signal in FIRES. The 2,000 mg/kg threshold warrants prospective dose-optimization trials using EEG-guided titration and alternative-agent strategies; however, it should not mandate arbitrary dose ceilings in this study. This threshold should prompt a mandatory review in FIRES and may serve as a readily calculable safety marker for any child receiving continuous propofol infusion.

## Data Availability

The original contributions presented in the study are included in the article/Supplementary material, further inquiries can be directed to the corresponding author.
