# Supplementary material for: Metabolism-corrected propofol exposure intensity and long-term intelligence quotient in pediatric febrile infection-related epilepsy syndrome: a retrospective cohort study
Source: Front Med (Lausanne). 2026 Feb 12;13:1747795. doi: 10.3389/fmed.2026.1747795 (PMC12935992; doi:10.3389/fmed.2026.1747795)
Supplement: Supplementary file 1 [file Table_1.docx]

****Supplementary Table 1.**** Interaction effect and additional robustness checks.

| **Analysis** | **β per 100 mg/kg DR** | **95 % CI** | **p-value** |
| --- | --- | --- | --- |
| Primary IPTW estimate | −0.41 | −0.68 to −0.14 | 0.003 |
| DR × age interaction (per 1-year increase) | −0.08* | −0.15 to −0.01 | 0.03 |
| Leave-one-out (n = 74 iterations) – β range | −0.44 to −0.38 | — | — |
| Quantile-g regression |  |  |  |
| 25th percentile | −0.39 | −0.67 to −0.11 | 0.007 |
| 50th percentile | −0.41 | −0.69 to −0.13 | 0.004 |
| 75th percentile | −0.43 | −0.71 to −0.15 | 0.003 |
| Replace PELOD-2 with P-MOD score | −0.40 | −0.67 to −0.13 | 0.004 |

*Interaction term: change in slope per one-year increase in age at onset.
